# Potential of Bike Sharing During Disruptive Public Health Crises: A Review of COVID-19 Impacts

**DOI:** 10.1177/03611981231160537

**Published:** 2023-04-11

**Authors:** João Filipe Teixeira, Cecília Silva, Frederico Moura e Sá

**Affiliations:** 1 Research Center for Territory, Transports and Environment (CITTA), Faculty of Engineering of the University of Porto, Porto, Portugal; 2 Centre for Studies in Governance, Competitiveness and Public Policies, University of Aveiro, Aveiro, Portugal

**Keywords:** pedestrians, bicycles, human factors, bicycle transportation, bike sharing

## Abstract

With public transport (PT) continuing to be negatively affected by the coronavirus pandemic and private car usage surging, alternative modes need to be considered. In this study, we review the available evidence (from academic and gray literature sources) on the performance of bike sharing systems (BSSs) during COVID-19 around the world, with the goal of assessing their potential contribution to improving the resilience of transport systems during pandemics and similar disruptive events. We found BSS usage followed a decrease-rebound pattern, with BSSs overall sustaining lower ridership declines and faster recoveries compared with PT. During lockdowns especially, the average duration of BSS trips increased, following a rise in casual users and leisure trips, while commuting trips decreased. Evidence has also been found for a possible modal shift from some PT users to BSSs, with a decline in the share of multimodal trips conducted between PT and BSSs. Bike sharing is perceived as safer than other shared modes (e.g., PT, taxis, and ride-hailing/sharing) but as having a higher infection risk than personal modes (e.g., private car, walking, and personal bike). Moreover, the BSS was an important transport alternative for essential workers, with several operators providing waivers especially to healthcare staff, leading to ridership increases near healthcare facilities and in deprived neighborhoods. Findings from this research support policies for promoting bike sharing, namely through fee reductions, system expansions, and symbiotic integration with PT, as BSSs can increase the sustainability and resilience of transport systems during disruptive public health events like COVID-19.

The ever more likely scenario of COVID-19 becoming endemic will have profound consequences (*1*, *2*), particularly in relation to mobility, which was heavily affected by the recent pandemic (*2*). Public transport (PT) in particular, has been greatly affected, suffering record-breaking ridership drops (*3*), which are still recovering 2 years after the beginning of the pandemic (*4*). The potentially permanent ridership loss of PT is likely to have severe repercussions, specifically concerning the pressing need for decarbonization (*5*) as the transport sector is responsible for around one quarter of all CO_2_ emissions (*6*), with car travel being the main source (*7*). Thus, with one of the most sustainable alternatives to car usage facing the prospect of long-term decline, additional modes of transport are urgently needed.

Among the potential alternatives to PT and car use are bike sharing systems (BSSs). Characterized by providing low-cost, short-term renting of bikes, either at stations (docked BSSs) or within operational areas (free-floating or dockless BSSs), BSSs have rapidly increased in popularity, with systems currently operating in more than 1,000 cities across the world (*8*). Bike sharing has already been shown to be a travel-time- and cost-competitive mode, capable of complementing PT and reducing car use (*9*). Furthermore, by avoiding the ownership inconveniences associated with a personal bike (e.g., purchase cost, the need for secure parking) and taking into consideration the benefits (e.g., greater trip flexibility), BSSs have the potential to increase cycling usage rates at the city level (*9*, *10*). For instance, BSSs are associated with reversing the cycling decline in China (*11*) and, even before COVID-19, BSSs have been shown to increase transport-system resilience by providing an alternative mode during subway strikes (*12*).

Therefore, it is paramount to understand how COVID-19 has affected the operation of BSSs to gauge their potential contribution to increasing the resilience and sustainability of urban transport systems, particularly during disruptive public health crises. As such, the main objective of this study was to review and synthesize the available research published during the first 2 years of the COVID-19 pandemic concerning bike sharing. We sought to answer the following questions:

- To date, what have been the main impacts of COVID-19 on bike sharing?
- How have BSSs performed in comparison to other modes during the pandemic?

This article is structured as follows. In the next section, the main literature search strategy (including searched databases, selection criteria, and keywords used) is detailed. This is followed by presentation of the COVID-19 impacts on BSSs. The article ends with the main research findings, detailing the policy implications and providing recommendations for policy makers wanting to make transport systems more resilient to pandemics by taking advantage of BSSs.

## Methodology

**Figure 1.**
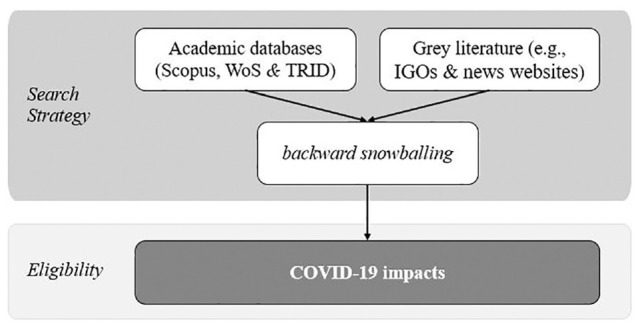
Diagram of the literature search strategy employed. *Note*: WoS = Web of Science; TRID = Transport Research International Documentation; IGOs = intergovernmental organizations.

**Figure 2.**
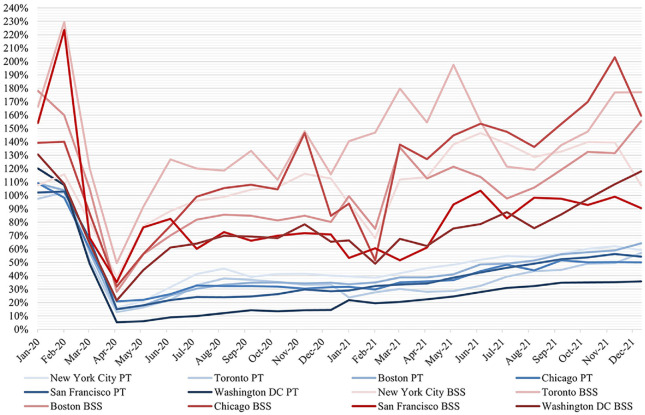
Monthly ridership difference for bike sharing systems (BSSs) (in red) and public transport (PT) (in blue) throughout 2020 and 2021 comparatively to 2019 (month-by-month comparison) in six North American cities. *Note*: Ridership was obtained from operators’ publicly available databases. PT ridership data were not available in New York City for January or February 2020. Websites were consulted on April 1, 2022. Boston (BSS: https://www.bluebikes.com/system-data; PT: https://mbta-massdot.opendata.arcgis.com/search?tags=ridership); Chicago (BSS: https://ride.divvybikes.com/system-data; PT: https://www.transitchicago.com/data/); New York City (BSS: https://ride.citibikenyc.com/system-data; PT: https://new.mta.info/coronavirus/ridership); San Francisco (BSS: https://www.lyft.com/bikes/bay-wheels/system-data; PT: https://www.sfmta.com/reports/muni-ridership-average-weekday-ridership); Toronto (BSS: https://ckan0.cf.opendata.inter.prod-toronto.ca/tr/dataset/bike-share-toronto-ridership-data; PT: https://open.toronto.ca/dataset/toronto-s-dashboard-key-indicators); Washington, D.C. (BSS: https://ride.capitalbikeshare.com/system-data; PT: https://www.wmata.com/initiatives/ridership-portal/).

Selection of the studies included in this review involved three stages (Figure 1). We started by searching for academic literature (articles published in peer-reviewed journals and conference papers with peer-reviewed proceedings) in Scopus, Web of Science (WoS) and the Transport Research International Documentation databases, using search word combinations of bike sharing and COVID-19. For the Scopus and WoS databases the specific search terms used were: (“coronavirus” OR “COVID-19” OR “Covid 19” OR “covid19” OR “SARS-CoV-2”) AND (“bike sharing” OR “bicycle sharing” OR “bike share” OR “bikeshare” OR “shared bicycle*” OR “bikesharing” OR “shared bike*” OR “public bicycle*” OR “public bike*”). Next, in light of COVID-19 initially being a novel coronavirus, coupled with the lengthy process until a peer-reviewed article is published, we also examined gray literature from BSS operators, public agencies, intergovernmental organizations, and news agency websites. Gray literature sources can provide valuable contributions to a literature review, especially for emerging topics such as bike sharing (*9*, *13*) and the pandemic (*14*). Lastly, backward snowballing (i.e., examining the references cited in the studies reviewed [*15*] in both sets of data sources) was undertaken. As a primary inclusion criterion, the studies had to have analyzed the impacts of COVID-19 on bike sharing. Secondary data on BSSs and PT ridership were also collected directly from operators’ websites. Specifically, we searched for cities in which ridership data on both BSSs and PT during COVID-19 were available in English. In total, six case studies, all from North America, were included (Figure 2).

The literature, collated between December 29, 2021 and March 31, 2022, was limited to studies written in English. The initial selection generated a total of 90 studies (58 peer-reviewed and 32 gray literature sources), with the final number of studies included in the review totaling 60 (42 peer-reviewed and 18 from gray literature). Most studies were published in 2021 and refer to the first COVID-19 wave of 2020. The review includes studies from across the world, but mainly Europe (particularly from the UK, Italy, Switzerland, Greece, and Portugal), North America (mostly from the United States), and Asia (especially China and South Korea). Furthermore, the majority of peer-reviewed studies evaluated the COVID-19 impacts on BSSs, with most research analyzing ridership data provided by the BSS operators, sometimes coupled with data from open access sources such as land use, points-of-interest, COVID-19 cases, and ridership from other modes of transport like PT. A few peer-reviewed studies conducted travel behavior surveys, either aimed at the general population in which bike sharing was one of the modes evaluated or specifically targeted at BSS users. Conversely, most gray literature focused on the measures implemented during COVID-19 by either operators or public authorities that affected BSSs. Lastly, a multitude of methods were applied to analyze the COVID-19 effects on BSSs, ranging from descriptive statistics and group comparison statistical tests (e.g., *t-*tests) to more complex methods, such as multivariate regressions, difference-in-difference models, and machine learning techniques.

## COVID-19 Impacts

We divided the main COVID-19 effects on bike sharing into six categories: ridership, length and duration of trips, trip purposes and travel patterns, relationship with PT, profile of BSS users, and attitudes. We start by presenting the main findings in Table 1. Each category is then explored in depth in the following subsections.

**Table 1.** Summary of the Main COVID-19 Impacts

Category	Main findings	References
Ridership	BSS ridership during COVID-19 followed a decrease-rebound pattern, with ridership severely declining during lockdowns, but quickly recovering when mobility restrictions were lifted, reaching and even surpassing prepandemic levels. The COVID-19 impact on ridership led to a decrease in bike fleets and even the closure of some systems, especially in the beginning of the pandemic. However, after ridership levels started to recover, BSSs reopened again, with some even expanding the number of stations and bikes. Compared with other modes, BSSs have generally sustained lower ridership drops and experienced faster recoveries, particularly when compared with PT systems.	(*8, 16–31*)
Length and duration of trips	The average distance and duration of BSS trips increased, especially during lockdowns.	(*18, 25–27, 31–38*)
Trip purposes and travel patterns	There was an overall increase in the share of leisure trips, whereas commuting trips fell in number. Residential and outdoor areas (such as parks) increased in popularity.	(*19, 25, 27, 33, 34, 37–42*)
Relationship with public transport	A change in the relationship between BSSs and PT from complementarity to competitivity was evidenced, with a decline in the share of BSSs+PT multimodal trips and evidence of a possible modal shift from PT to BSSs.	(*25, 28, 31, 32, 39, 43–45*)
Profile of BSS users	The share of casual users increased, whereas long-term members declined. Bike sharing was sought after by essential workers as a transport alternative during COVID-19, with ridership increases at BSS stations near healthcare facilities and within deprived neighborhoods.	(*19, 25–27, 32, 34, 38, 39, 46, 47*)
Attitudes	BSSs were perceived as having a lower infection risk than other shared modes (e.g., PT, taxis, and ride-hailing/sharing) but riskier than using personal modes (e.g., private car, walking, and personal bike). There was an increase in the importance of using BSSs to avoid PT and for social distancing purposes. Measures implemented to reduce infection risk and publicizing such efforts increased trust in bike sharing.	(*28, 45, 48–56*)

*Note*: BSSs = bike sharing systems; PT = public transport.

### Ridership

BSS ridership during the pandemic seemed to follow a decrease-rebound pattern. Like most modes of transport, BSS usage rapidly plummeted when the number of COVID-19 cases started to increase and restrictions on mobility came into effect—trends being observed across the world, for example, in China (*18*, *57*), Europe (*28*, *30*) and North America (*25*, *31*). For instance, New York City’s BSS, Citi Bike, had registered a 71% ridership drop by the end of March 2020 compared with prepandemic levels registered early in the month (*26*, *31*). Likewise, in Beijing (China) COVID-19 triggered a 65% ridership reduction in the city’s BSS from January 1 to March 1, 2020 compared with the same period in 2019 (*57*). However, after the number of COVID-19 cases had started to fall and mobility restrictions were gradually lifted, BSS usage quickly recovered, with several cases reaching and sometimes even surpassing prepandemic ridership levels. Again, similar patterns were detected across different continents, including in Europe (*27–29*, *58*), North America (*16*, *24–26*), and South Korea (*17*, *43*). For instance, Citi Bike ridership started to improve in early April, fully recovering its ridership to 2019 levels by September 2020 (*26*). Meanwhile, in Seoul (South Korea), which did not implement a full lockdown, the daily ridership of the city’s public BSSs between January and March 2020 was double that registered for the same period in 2019 (*17*).

These ridership declines registered at the beginning of the pandemic, led several BSSs to suspend their services, with some operators decreasing their shared bike fleets and even permanently closing. For example, on March 2020, the city of Austin, TX, ordered all shared mobility operators (including BSSs) to reduce their fleets by 10% owing to the reduction in demand (*20*). The Meddin Bike-Sharing World Map (*8*) reported that 2020 was the first year since they had started recording the number of BSSs in operation (back in 2007) in which the number of BSSs closing had surpassed the number of systems launched (194 versus 115), with most closures occurring in the United States (120 BSSs closed). However, after the initial shock and BSS ridership levels started to recover, most BSSs reopened again, with some even expanding the number of stations and bikes (*59*), including opening new BSS stations near hospitals to improve access for healthcare workers (*21*, *22*). By mid-2021 the number of new BSSs had surpassed the number of systems closing (*8*). Furthermore, North American Bikeshare & Scootershare Association (*40*) reported that the number of available shared bikes in North America had actually increased following a surge in e-bikes, which increased from 12,000 in 2019 to 23,000 in 2020 (whereas the number of shared e-scooters decreased by 31%), with 75% of the suspended systems being reopened by the end of the year. Similarly, in May 2020, China’s largest dockless BSS operator announced the deployment of hundreds of thousands of shared e-bikes because of a boom in e-bike demand (*23*).

More remarkably, evidence suggests that BSSs have sustained lower ridership drops and faster recoveries than other modes, particularly PT (*24–26, 29–31, 38, 39, 60*) but also in some cases in relation to private car (*25*, *43*), taxis (*19*), walking (*25*), personal cycling (*30*), and e-scooter sharing (*40*). For example, the city of Chicago, IL (*25*) compared the traffic levels of the city’s BSS (named Divvy), PT, driving, and walking between March and July 2020 to a baseline volume on January 13, 2020 (prepandemic). The authors found Divvy had registered the “highest recovery speed and greatest recovery magnitude” of all modes, with BSS ridership reaching 284% in July 2020; for the same period driving and walking had increased 137.5% and 131.6%, respectively. In contrast, PT was still 45.8% of the baseline volume in July 2020 (*25*). Additional evidence can again be found in New York. While at the height of the first COVID-19 wave in March 2020, Citi Bike was suffering a 71% ridership drop (*19*, *26*, *31*), the subway system had a 91% drop (*26*, *31*), whereas the city’s yellow taxi service declined by 96% (*19*). Moreover, after lockdown easing in June 2020, the BSS ridership drop had decreased to 28% (fully recovering during the month of September 2020) whereas the subway and taxi services were still suffering from an 83% and 94% ridership decline, respectively (*19*, *26*). In fact, Lei and Ozbay revealed that although before COVID-19 the number of taxi trips was much higher than Citi Bike, during the pandemic Citi Bike actually surpassed taxi ridership, with the gap continuing to widen until the end of the study (June 2020) (*19*). These findings were supported by Ku et al., who conducted a similar study in Seoul (*43*). The authors found that while both PT and car usage decreased as COVID-19 cases surged, BSS ridership increased, registering a 121% ridership level in June 2020 compared with 2019 levels, whereas car and PT levels were 100% and 71.5%, respectively (*43*). Similarly, in Nanjing (China), during lockdown metro ridership fell 95% compared with prepandemic levels, however, ridership of the city’s docked and dockless BSSs dropped 72% and 82%, respectively (*38*).

We further explored the resilience of BSSs to the pandemic by assessing the difference in the average monthly ridership of six BSSs in North America (Boston, Chicago, New York City, San Francisco, Toronto, and Washington D.C.) throughout 2020 and 2021 to 2019 levels (month-by-month comparison), and comparing this with the cities’ PT systems. The disaggregated values for each city are presented in Figure 2. We can observe that the ridership of both BSSs and PT drastically declined during the first COVID-19 wave, dropping to their lowest points during April 2020, with ridership levels starting to recover after this initial wave. There were further ridership drops during the next waves but with lower magnitudes. However, the figure also reveals that the BSS ridership changes were always more positive than the corresponding PT changes. More importantly, we can observe that by December 2021 (2 years after the pandemic began) PT ridership was still only around 60% of prepandemic levels, whereas most BSSs had already recovered with their ridership levels closer to and in most cases even above prepandemic levels.

### Length and Duration of Trips

The average distance and duration of BSS trips generally increased during COVID-19, particularly during lockdowns, with an increase in the proportion of long trips taken while that of short trips declined. In London, during the first full lockdown, Li et al. noted that the city’s BSS ridership fell significantly except for long duration trips (i.e., trips longer than 30 to 60 min) (*27*), whereas in Beijing, Shang et al. observed BSS average trip durations during lockdown increasing from 22.5 to 26.5 min (*18*). Likewise, in New York City, Citi Bike’s average trip duration increased from a 12 or 13 min to a 19 or 20 min daily average by the end of March 2020 (*31*, *39*), with the ratio of long duration trips doubling (*26*), and consistently staying above 2019 levels for the rest of the year (*26*, *34*). Similar trends were observed in Boston (*34*), Chicago (*25*, *34*), Greece (*35*), Košice (Slovakia) (*36*), Montreal (Canada) (*37*), Nanjing (*32*, *38*), Seoul (*41*), and Zurich (Switzerland) (*33*). There are two main reasons behind this increase in the duration/length of the BSS trips, which will be explored in depth in the next two subsections: on the one hand an increase in leisure trips, and on the other, a possible modal shift of some PT users to bike sharing.

### Trip Purposes and Travel Patterns

COVID-19 changed the purposes of BSS trips, leading to an overall increase in leisure trips and a reduction in commuting trips. Several studies support this assertion. Firstly, BSS trips conducted during nonworking days were less affected than those conducted during weekdays (*25*, *33*, *34*, *38*, *40*). For example, in Chicago, whereas before COVID-19 most Divvy trips were conducted on weekdays, during the pandemic that trend shifted, with weekends now registering greater ridership levels (*25*). A similar pattern of increased usage on weekends was also observed in Nanjing (*38*) and New York City, where weekend BSS ridership surpassed 2019 levels (*19*). Secondly, whereas before COVID-19 BSS travel patterns were characterized by a two-peak (morning and evening) daily commuting pattern, the pandemic caused a decline in those peaks (*25*, *27*, *33*, *37*, *38*), in some cases changing to a one-peak leisure pattern (*25*). Thirdly, the number of roundtrips (i.e., trips that start and end at the same BSS station) increased, for instance, Chicago’s Divvy roundtrip share grew from 4.7% to 12.7% (*25*).

The increase in the leisure-oriented nature of bike sharing during COVID-19 was further supported by changes in the main origins and destinations of BSS trips, with the share of trips near residential and outdoor areas increasing (*25*, *33*, *41*, *42*). For instance, Li et al., through analyzing GPS trip data from 3,700 users of both docked and dockless bike sharing in Zurich, found the share of BSS trips near residential areas as well as grocery shops and outdoor activities (such as parks) increased for all types of BSS, whereas the share of trips near workplaces declined (*33*). Similarly, in Chicago, Divvy stations near residential areas and outdoor activities experienced lower ridership decreases during lockdown and recovered earlier than other stations (*25*). Additional COVID-19 impacts on the origin–destination of BSS trips were identified. BSS stations located in city centers experienced higher ridership drops, whereas stations located in peripherical zones were less affected (*25*, *33*, *39*); this could be attributed to a reduction in travel demand both for commuting and other activities that were heavily restricted during COVID-19. Changes in BSS trips near PT stations were also observed but with conflicting reports. While in both London (*27*) and Seoul (*41*) a decrease in BSS trips near PT stations was observed, in Chicago regions with higher PT ridership levels registered lower BSS ridership reductions (*25*). Regardless of this, these results seem to indicate a decrease in BSS commuting trips conducted in combination with PT either as a result of stay-at-home orders and/or BSS users replacing multimodal BSS+PT trips with bikes for the whole journey. The next subsection will explore in depth the COVID-19 impacts on the relationship between BSSs and PT.

### Relationship With Public Transport

As stated, BSSs have proved more resilient than PT during the pandemic, showing lower ridership drops during stay-at-home orders and faster recovery rates when mobility restrictions were lifted. In the following, we further explore how COVID-19 has affected the relationship between bike sharing and PT.

The pandemic seems to have accentuated the competitiveness between BSSs and PT. Before COVID-19, bike sharing was an important feeder to PT (*9*), particularly rail transport (*61 [Bibr bibr62-03611981231160537]*–*63*). For example, the Dutch rail company implemented a bike sharing/rental system, OV-fiets, specifically to boost train ridership, which led to an increase in the share of bike–train users from 30% to 50% (*64*). At the same time, bike sharing has the potential to replace PT trips, particularly in overcrowded systems (*9*) and during disruptive events such as PT strikes (*12*).

With the emergence of the pandemic, modal shift from PT to BSSs seems to have intensified (*25, 28, 31, 32, 39, 43, 44*). For example, in New York City, the subway/BSS ridership ratio during March 2020 was found to have decreased more significantly inside the subway’s catchment areas (*31*). Coupled with an increase in Citi Bike’s average trip duration (even during weekdays), this pointed to a possible modal shift of some subway users to BSSs (*31*). Furthermore, while subway ridership fell uniformly throughout all of New York City’s boroughs, Citi Bike ridership in some boroughs actually increased during March 2020, particularly at BSS stations alongside subway lines (*39*). Such findings were supported by Ku et al., who estimated that the modal share of BSSs in Seoul increased from 0.3% to 0.5%, whereas, as of June 2020, the modal share of Seoul’s metrorail had declined from 1.3% to 0.9% (*43*).

Further evidence can be found in two surveys of BSS users undertaken in 2020. First, a cross-country survey aimed at users of UK’s BSSs found that 29% of respondents reported using BSSs as an alternative to PT as a result of COVID-19 (*45*). A similar survey conducted with BSS users in Lisbon (Portugal) revealed that PT had been surpassed by GIRA (Lisbon’s BSS) as the main mode of transport, with 71% of respondents (versus 48% before COVID-19) at the time considering using GIRA to avoid PT as an “important” or “very important” motivation (*28*). Interestingly, this study also explored GIRA’s modal shift dynamics by asking the respondents which mode of transport they would use if the BSS was not available to them both before and during COVID-19 (*28*). The survey revealed that even though PT was the most replaced mode in both periods, its share had actually decreased (while the proportion of replaced private car, walking, and personal bike had increased). Such results led to the hypothesis that some respondents no longer considered PT as a transport alternative because of fears of infection, and would now choose other modes of transport if BSSs were not available (*28*). We will further explore this issue by delving into the attitudes and motivations of BSS users in the next subsections.

Several studies have concurrently found evidence of a decline in the share of multimodal trips conducted between BSSs and PT, while the share of BSS single trips has increased (*28*, *32*, *45*). In the aforementioned UK survey the percentage of BSS trips conducted in combination with bus and train decreased from 23% and 35% to 9% and 10%, respectively (*45*), whereas in Lisbon, the share of BSSs+PT trips declined from 24.9% to 17.2% (*28*). These results are further supported by Chen et al. who, using smart card data in Nanjing, found that the share of BSS–metro commuters had decreased from 44.2% (16.2% of all BSS users) to 36.1% (12.5% of all BSS users) (*32*). Such reductions in the share of multimodal trips between the two modes, accompanied by an increase in BSS single trips, further support the change on the relationship between BSSs and PT from complementarity to competitivity.

### Profile of BSS Users

Several authors have observed a reduction in the proportion of long-term BSS members (defined as BSS users with a monthly/yearly subscription), whereas the share of casual users has increased (*19*, *25*, *26*, *34*). For example, in Chicago the share of trips by Divvy members declined from 75% in 2019 to 56% in 2020, whereas the proportion of trips conducted by casual users significantly increased from 25% in 2019 to 44% in 2020 (*25*). As casual users tend to be more associated with using bike sharing for leisure purposes, whereas BSS members are more connected with commuting trips, such findings further support the previously discussed increase of BSS leisure-oriented trips during COVID-19.

When considering the COVID-19 impacts on the sociodemographic and economic profiles of BSS users, the literature diverges. On the one hand, two peer-reviewed studies of Nanjing found a reduction in the share of female as well as of younger users (*32*, *38*); this was attributed to women being more likely to be responsible for caring for children (who, because of the pandemic, had shifted to online classes) and younger cohorts being more likely to telework (*32*). However, studies from Western countries found an increase in the share of female users. In New York City the share of female BSS users increased from 23.6% to 36.0%, with women riding for longer durations (*65*). Moreover, an increase in usage by younger cohorts under the age of 35 was also noted (*65*). In Barcelona (Spain), Bustamante et al. observed that neighborhoods comprising a higher proportion of women increased their BSS use (*66*). A possible explanation for these findings can be found in the study conducted by Teixeira and Cunha who surveyed users of Lisbon’s BSS and explored differences in the travel behavior and attitudes between women and men before and during COVID-19 (*67*). The study discovered that before the pandemic, men were using BSSs more frequently than women, but during COVID-19 women started to use bike sharing as frequently as men. The authors rationalized this shift by suggesting that BSS trip purposes differed between genders, with men more likely to use BSSs for commuting while women were more likely to use BSSs for leisure and exercise. As the demand for commuting dropped significantly during the pandemic owing to government travel restrictions such as mandatory teleworking, leisure trips increased and the usage difference between genders disappeared (*67*).

Crucially, bike sharing has provided essential workers with a transport alternative during COVID-19. BSS stations near healthcare facilities like hospitals have increased in popularity in cities such as London (*27*), Nanjing (*38*), Boston, New York City, and San Francisco (*46*). Moreover, BSS stations located in neighborhoods comprising higher levels of socially disadvantaged groups (e.g., low-income households and minorities) were more resilient, with lower ridership drops (*25*, *39*) and faster recovery rates (*25*, *27*). Socially disadvantaged groups, which constituted a significant share of essential workers during COVID-19 (e.g., food retail workers), were more likely to commute even during lockdowns (*25*, *47*) and, faced with restrictions (and infection fears related to using PT), turned to BSSs as an affordable transport alternative. Dozens of BSSs across the world introduced discounted or free trips in the first months of the pandemic, which were particularly aimed at essential workers (*20, 28, 31, 39, 40, 47, 68, 69*), with some systems providing discounted or free trips for all users (*68*, *70*). Although there is still little evidence about the impact of such discount programs, specifically on overall BSS usage levels, they seem to have been well received with thousands of essential workers enrolling. For instance, more than 18,000 healthcare professionals joined the program in London (*71*) whereas in New York the Citi Bike Critical Workforce Membership Program, which in addition to healthcare staff also included PT employees, attracted more than 33,000 workers (*72*). Assessing its effects in New York, Pase et al. found that the program had had a significant impact on Citi Bike ridership, with trips conducted by essential workers subscribing to the program increasing from a residual share at its launch in March 2020 to representing 8.1% of all trips by June of that year (*39*). However, the fact that most of these discount programs were limited to healthcare staff raises equity issues, as they excluded other types of essential workers, particularly low-income groups such as food retail workers who were among those most in need of alternatives to PT (*47*).

### Attitudes

The pandemic has also changed the reasons for using bike sharing, with an increase in the importance of using BSSs to avoid PT and to ensure social distancing (*28*, *45*, *54*). The UK’s 2020 cross-country survey of BSS users found that the main reasons for (re)joining BSSs for 23% of respondents were the lockdown and restrictions on PT (*45*). In conducting a travel survey of GIRA users (Lisbon&rsquo;s BSS), Teixeira et al. classified the reasons for using bike sharing into five motivational factors (“service coverage & quality”, “personal interests & well-being”, “utility & competitiveness”, “social influence”, and “social discomfort”), ranking their importance before and during COVID-19 (*54*). The authors revealed that the motivations connected to COVID-19, specifically using GIRA to avoid PT and to socially distance (which the authors designated as the “social discomfort factor”) had increased in importance, being now only surpassed in importance by the motivations connected to the system’s coverage and quality (*54*).

In fact, an emerging trend from the literature points to bike sharing being perceived as having a lower COVID-19 infection risk than other shared modes, particularly PT, taxis, and ride-hailing/sharing, but riskier than using personal modes such as private car, walking, and personal bike (*28, 49–53*). For instance, shared bikes in Chicago were ranked as having the fourth highest perceived infection risk, after private cars, walking, and personal bicycles, but before PT, taxis, and ride-hailing services (*50*). The perceived safety of BSSs during COVID-19 is further supported by Lisbon’s GIRA users who largely quit or reduced their usage during COVID-19 because of a decrease in the requirement to travel (particularly commuting) rather than to infection fears (*28*).

Considering bike sharing as a safe mode during COVID-19 may have induced an increase in BSS usage rates (*49*, *55*). A survey of users of a small BSS in Thessaloniki (Greece) revealed that respondents with a more positive view of BSS safety during the pandemic were more likely to use them (*49*). Similarly, most respondents in a survey conducted by the consultancy McKinsey & Company in seven countries mentioned frequent disinfection (47%), social distancing (43%), and user health checks (31%) as measures that would increase their likelihood of using shared micromobility (including BSSs) (*56*). Such findings support the several measures implemented by BSS operators to reduce the infection risk from contaminated surfaces (e.g., through enhancing the cleaning and disinfection of shared bikes and other high-touch surfaces such as touchpads at the stations) and from contact with other users when picking up or dropping off bikes (e.g., disclosing crowding levels at BSS stations and recommending users wear a facemask) (*21*, *40*, *48*, *73*). However, Jobe and Griffin found that of the 11 U.S. cities they analyzed, only five BSS operators had publicized the sanitary and health measures taken to reduce the COVID-19 infection risk for their users (*48*). Moreover, a survey of the San Antonio BSS, revealed that only 57% of the users were aware of the actions taken by the operator to minimize the COVID-19 transmission risk (*48*). As such, BSS operators should widely publicize their efforts to reduce infection risk, as more effective communication of the infection prevention measures implemented could increase the perceived safety of BSSs, which in turn might lead to higher BSS usage.

## Conclusions

### Main Research Findings and Policy Insights

This review has synthesized the main impacts of COVID-19 on BSSs (summarized in Table 1). Table 2 presents recommendations for how policy makers and BSS operators could take advantage of BSSs to increase the resilience of transport systems during the current pandemic as well as during any future similar disruptive public health crises.

**Table 2.** Recommendations for Policy Makers and Operators for the Operation of Bike Sharing Systems During Disruptive Public Health Crises such as the Coronavirus Pandemic

Category	Measures	Aimed at
System expansion	Deploy additional BSS stations and shared bicycles (for instance, during reopening phases) near major trip generators (such as healthcare facilities and other destinations with higher demand during a pandemic) to reduce the pressure from PT services	Policy makers and operators
	Provide funding for BSS expansion into peripheral areas where transport alternatives are especially needed	Policy makers
	Introduce shared e-bikes as their longer range is especially suited to replacing PT and car trips	Policy makers and operators
COVID-19 infection risk reduction	Implement measures to reduce the infection risk of BSSs, such as enhancing cleaning and disinfection protocols, enforcing social distancing, as well as recommending users to disinfect their hands and wear a facemask	Operators
	Promote bike sharing as an alternative mode of transport capable of ensuring social distancing Publicize the health and safety measures implemented	Operators
Service and business models	Subsidize waivers/free trips for essential workers to include not only healthcare staff but more vulnerable groups such as food retail workers	Policy makers and operators
	Provide monthly/annual passes and/or long-term monthly rentals to attract former PT commuters to bike sharing	Policy makers and operators
	To compensate for revenue losses, particularly during lockdowns, BSS operators could forge partnerships with food-delivery companies (taking advantage of the heightened demand for home-delivery services such as take-out food)	Operators

*Note*: BSSs = bike sharing systems; PT = public transport.

Among the most important findings, we uncovered robust evidence for BSSs to substitute PT systems during the pandemic as well as its potential during similar disruptive public health crises. Firstly, BSSs overall suffered lower ridership drops and faster recoveries than PT, with several BSSs recovering and even surpassing prepandemic usage levels whereas PT ridership remained battered. Secondly, the share of multimodal trips combining BSSs and PT has declined, while at the same time the average trip duration of BSS trips has increased. Thirdly, bike sharing is considered to have a lower infection risk than PT, with users reporting choosing BSSs during COVID-19 to avoid PT and to maintain social distancing during their trips. This has important policy implications: at a time when PT systems are still experiencing lower ridership levels and car usage is rising (with all the consequent negative impacts), BSSs could potentially be an alternative. However, most BSSs in operation are too small to effectively provide an alternative to most PT users. For instance, New York’s Citi Bike, one of the largest BSSs outside of China, broke its daily ridership record on September 11, 2021, with 135,005 rides (*74*), which only corresponds to around 2.5% of the subway’s prepandemic average weekday ridership of 5.5 million trips (*31*). Expanding BSSs, which continue to be largely limited to city centers, could be a solution. For example, Beijing’s dockless BSSs have more than 11 million users, which corresponds to approximately half the city’s population (21.5 million inhabitants), registering over 1.5 million trips per day (*57*). The deployment of shared e-bikes could also be particularly useful as they increase the range of cycling trips, therefore, being especially suited for replacing PT and car trips.

We also found evidence of the importance of bike sharing for leisure activities during COVID-19. With most options for exercising closed during the height of the pandemic, particularly indoor facilities such as gyms and swimming pools, BSSs provided a means of exercising. Maintaining recommended physical activity levels has important benefits not only for physical health (e.g., maintaining a healthy weight) but also for mental health (e.g., minimizing depression and anxiety) (*75*), both of which were negatively affected by the pandemic (*76*, *77*).

Furthermore, bike sharing emerged as a valuable transport alternative for essential workers and other citizens who still had to travel during the height of the pandemic. BSS ridership near healthcare facilities and essential businesses (e.g., grocery stores) increased, with record numbers of essential workers subscribing to bike sharing, taking advantage of the discounted/free membership programs provided by several operators. With PT heavily restricted during COVID-19, bike sharing was especially important to some of the most vulnerable groups, with BSS stations in deprived neighborhoods increasing in importance. However, restriction of the discounted/free membership programs to mostly healthcare staff excluded low-income essential workers (e.g., working in food retail), thereby depriving some of the groups most in need of alternatives to PT during the pandemic. Bike sharing was known to have equity issues before the pandemic, largely because these systems tend to be concentrated in city centers where the density of both population and activities makes them more profitable (*9*), leading operators to disregard more peripheral areas. In that sense, the waiver programs implemented during the pandemic were a lost opportunity to minimize some of these inequity impacts, as public funding to subsidize BSS trips in deprived neighborhoods could have incentivized BSSs to expand their operations to less profitable areas.

### Research Needs

This review opens several research paths. Firstly, it is paramount to further investigate the effects of COVID-19 on modal shift dynamics. It is especially important to assess the potential for BSSs to provide an alternative to PT, specifically, through quantifying the actual modal shift from PT to BSSs registered during COVID-19. Equally as important will be to analyze the potential of BSSs to ameliorate (and identify to what extent this might occur) a surge in private car usage, either by directly replacing car trips or by avoiding a modal shift from PT users to the private car. Replicating research designs applied in prepandemic modal shift studies could offer potential paths (*9*, *78*).

The methods used in qualitative studies, for example, interviews and focus groups, will also be necessasry, as such research designs would allow a more in-depth understanding of individual behavior changes in relation to the use of bike sharing: why users chose BSSs as their mode of transport during the pandemic when other alternatives were available. Surveys aimed at gauging the attitudes of the broader population (i.e., nonusers) toward BSSs during COVID-19 would also be useful to estimate the potential for bike sharing at a city level. Likewise, other sources like geotagged twitter-, cellular phone-, and location-based service data could help further investigate the pandemic impacts on travel patterns, as most of the available studies used surveys or trip data from the BSS companies.

Studies aimed at exploring the impact of the pandemic on the socioeconomic and demographic profiles of BSS users are required. For instance, we speculate that the divergency of impact on gender found between China and Western case studies could be related to their different cycling contexts. In China, cycling is much more widespread leading to women cycling as often as men, with the share of female and male BSS users in Nanjing before COVID-19 being roughly equal (*38*)—similar to other mature cycling contexts such as the Netherlands (*79*). However, the three Western case studies that examined gender differences during COVID-19 are all examples of low-use cycling contexts, in which women are much less likely to use bike sharing (as well as cycling) (*9*, *80*), because of women’s greater safety concerns (*79*, *81*). The increase in the share of female users observed in the Western case studies could be related to the several measures implemented by public authorities during the pandemic aimed at promoting cycling and walking, such as car-free streets and segregated cycling lanes (*82*), which in turn could have made BSSs (and cycling) more attractive to women. A possible future research path could, thus, be to analyze to what extent these policy responses have created safer cycling environments and, in turn, to confirm whether such environments have actually led to more equitable BSS usage.

Lastly, as the published literature on COVID-19 and bike sharing grows, future reviews should rely more on peer-reviewed studies instead of gray literature sources, while also introducing quality assessment tools such the Risk of Bias Assessment and the Evaluation of Certainty of Evidence (*83*) to increase the confidence on the obtained results.
